# The Different Insulin-Sensitising and Anti-Inflammatory Effects of Palmitoleic Acid and Oleic Acid in a Prediabetes Model

**DOI:** 10.1155/2022/4587907

**Published:** 2022-09-13

**Authors:** Denisa Miklankova, Irena Markova, Martina Hüttl, Barbora Stankova, Hana Malinska

**Affiliations:** ^1^Centre for Experimental Medicine, Institute for Clinical and Experimental Medicine, Prague, Czech Republic; ^2^First Faculty of Medicine, Charles University, Prague, Czech Republic

## Abstract

**Introduction:**

Monounsaturated fatty acids (MUFA) are understood to have therapeutic and preventive effects on chronic complications associated with type 2 diabetes mellitus (T2DM); however, there are differences between individual MUFAs. Although the effects of palmitoleic acid (POA) are still debated, POA can regulate glucose homeostasis, lipid metabolism, and cytokine production, thus improving metabolic disorders. In this study, we investigated and compared the metabolic effects of POA and oleic acid (OA) supplementation on glucose and lipid metabolism, insulin sensitivity, and inflammation in a prediabetic model, the hereditary hypertriglyceridemic rat (HHTg). HHTg rats exhibiting genetically determined hypertriglyceridemia, insulin resistance, and impaired glucose tolerance were fed a standard diet. POA and OA were each administered intragastrically at a dose of 100 mg/kg b.wt. for four weeks.

**Results:**

Supplementation with both MUFAs significantly elevated insulin and glucagon levels, but only POA decreased nonfasting glucose. POA-treated rats showed elevated circulating NEFA associated with increased lipolysis, lipoprotein lipase gene expression, and fatty acid reesterification in visceral adipose tissue (VAT). The mechanism of improved insulin sensitivity of peripheral tissues (measured as insulin-stimulated lipogenesis and glycogenesis) in POA-treated HHTg rats could contribute increased circulating adiponectin and omentin levels together with elevated *FADS1* gene expression in VAT. POA-supplemented rats exhibited markedly decreased proinflammatory cytokine production by VAT, which can alleviate chronic inflammation. OA-supplemented rats exhibited decreased arachidonic acid (AA) profiles and decreased proinflammatory AA-derived metabolites (20-HETE) in membrane phospholipids of peripheral tissues. Slightly increased *FADS1* gene expression after OA along with increased adiponectin production by VAT was reflected in slightly ameliorated adipose tissue insulin sensitivity (increased insulin-stimulated lipogenesis).

**Conclusions:**

Our results show that POA served as a lipokine, ameliorating insulin sensitivity in peripheral tissue and markedly modulating the metabolic activity of VAT including cytokine secretion. OA had a beneficial effect on lipid metabolism and improved inflammation by modulating AA metabolism.

## 1. Introduction

Evidence suggests that lipids, in particular fatty acids (FA), may play an important developmental role in the pathogenesis of metabolic syndrome (MS), T2DM, and its complications and also act as a link between adipocytes and whole-body metabolism [1]. The FA composition of dietary fats can influence different metabolic processes in the body including energy balance, chronic inflammation, glucose tolerance, and insulin action [2]. Specific classes of FAs vary considerably in their metabolic effects. High intake of a diet rich in saturated fatty acids (SFA) has been shown to be proinflammatory and deleterious to cardiovascular functioning with the potential to promote low chronic inflammation, insulin resistance (IR), and cardiovascular disease [3]. In contrast to their saturated counterparts, elevated intake of monounsaturated fatty acids (MUFA) can be associated with anti-inflammatory effects and improved IS. MUFA can improve blood lipid profiles, IS, and glucose control [4], while protecting *β* cells against apoptosis [5]. However, some of their effects are poorly understood. Despite the reported beneficial effects of MUFA, an increase in plasma MUFA levels can adversely influence incidence of T2DM [6]. The evidence indicates that there are differences in the metabolic effects between individual MUFA, but only a few studies thus far have compared the effects of individual MUFA.

The results of recent human and animal studies highlight the beneficial effects of POA (C16:1n7) on IS and glucose homeostasis. POA is one of the most abundant omega-7 MUFAs and is predominantly endogenously synthesised from palmitic acid (PA) by the stearoyl-coenzyme A desaturase 1 (SCD-1) enzyme [7]. Animal and human studies have shown that circulating levels of POA are associated with improved IS, glucose homeostasis, lipid metabolism, and inflammatory cytokine production [8]. Other studies, however, report that increased levels of POA may be associated with higher risk of T2DM and the development of IR [9].

OA (C18:1n9), the most abundant MUFA in the human diet, has been shown to mediate the protective effects on IS in a number of ways that are different to POA. OA can improve IS through adiponectin elevation and gene upregulation, support FA oxidation, increase inflammatory mediators, and modulate the transfer of glucose to muscles [10, 11]. However, the differences in the mechanisms and beneficial effects of POA and OA remain unclear.

It is also not clear how these MUFAs influence adipocyte secretion by VAT. It has been shown that POA can act not only as a lipokine but also as a regulatory molecule capable of modulating insulin signaling, inflammatory processes, and various metabolic pathways in different tissues [12]. Unfortunately, the exact mechanisms behind these activities and, indeed, whether OA may act in a similar manner are not known.

To address the deficit in the knowledge, our primary aim was to investigate and compare the effects of POA and OA on IS in peripheral tissue, chronic inflammation, and glucose and lipid metabolism in a prediabetic rat model. Our secondary aim was to determine the potential roles of these acids as lipokines. Our rat strain was characterised by genetically determined hypertriglyceridemia, IR, and low-grade inflammation in the absence of obesity and fasting hyperglycemia [13].

## 2. Materials and Methods

### 2.1. Animals and Diet

All of the experiments were performed in agreement with the Animal Protection Law of the Czech Republic (311/1997), which is in compliance with European Community Council recommendations (86/609/ECC) for the use of laboratory animals, and approved by the Ethics Committee of the Institute for Clinical and Experimental Medicine. The study was performed on six-month-old male HHTg rats (provided by the Institute for Clinical and Experimental Medicine, Prague, Czech Republic) as a nonobese prediabetic model. Rats were kept in temperature- (22°C) and humidity-controlled conditions under a 12 h/12 h light/dark cycle with free access to a standard diet (Altromin, maintenance diet for rats and mice, Germany) and drinking water. Rats were randomised into three groups: control (C), POA, and OA. POA (*cis*-16:1n7, Sigma-Aldrich) or OA (*cis*-18:1n9, Sigma-Aldrich) was administrated intragastrically at a dose of 100 mg/kg b.wt. daily for four weeks. For the control group, PBS 0.01 M solution (pH 7.4) was intragastrically administrated at the same dose. After POA supplementation, the proportion of *cis*-16:1n7 in serum NEFA markedly increased (64%, *p* < 0.01). After OA supplementation, the proportion of *cis*-18:1n9 in serum NEFA markedly increased (25%, *p* < 0.001).

At the end of the experiment, rats were sacrificed after light anaesthetisation (zoletil 5 mg/kg b.wt.) in a postprandial state. Aliquots of serum and tissue samples were collected and stored at -80°C for further analysis.

### 2.2. Analytical Methods/Biochemical Analysis of Serum and Tissues

Serum levels of triglycerides (TG), glucose, NEFA, and total/HDL cholesterol were measured using commercially available kits (Erba Lachema, Brno, Czech Republic, and Roche Diagnostics, Mannheim, Germany).

Serum insulin, glucagon, hsCRP, IL-6, and omentin concentrations were determined using Rat ELISA kits (Mercodia AB, Uppsala, Sweden; BioVendor, Brno, Czech Republic; MyBioSource, San Diego, CA, USA; BlueGene, Shanghai, China). Concentrations of MCP-1, TNF*α*, leptin, resistin, HMW adiponectin, 14,15-epoxyeicosatrienoic acid (14,15-EET), and 20-hydroxyeicosatetraenoic acid (20-HETE) in serum and tissue homogenates were measured using rat ELISA kits (Invitrogen, Vienna, Austria; BioVendor, Brno, Czech Republic; MyBioSource, San Diego, CA, USA).

For the oral glucose tolerance test (OGTT), blood glucose was determined after a glucose load (300 mg/100 g b.wt.) administered intragastrically after overnight fasting. Blood was drawn from the tail before the glucose load at 0 min and then at 30, 60, 120, and 180 min thereafter.

For determination of TG in muscles, samples were extracted in chloroform/methanol. The resulting pellet was dissolved in isopropyl alcohol, with TG content determined by enzymatic assay (Erba-Lachema, Brno, Czech Republic). To determine diacylglycerols (DAG) in tissues, samples were extracted in dichloromethane/methanol. The resulting pellet was dissolved in isopropyl alcohol and isolated by thin-layer chromatography (TLC). The content of separated DAG was determined by enzymatic assay (Erba-Lachema, Brno, Czech Republic).

As a marker of skeletal muscle (glycogenesis) and adipose tissue insulin sensitivity (lipogenesis), basal and insulin-stimulated glycogen or lipid synthesis was determined *ex vivo* in the isolated musculus soleus or epididymal fat pad by measuring the incorporation of ^14^C-U glucose into glycogen or lipids as described previously [14]. In epididymal adipose tissue (EAT), basal and adrenaline-stimulated lipolysis was measured *ex vivo* based on the release of NEFA into the incubating medium. Reesterification of FAs was calculated as the NEFA/glycerol ratio (Ranox, Antrim, United Kingdom) in the incubating medium.

### 2.3. Fatty Acid Profile and Fatty Acid Desaturase Activity

FA levels were reported as a percentage of the total FAs. For determination of FA composition in muscles or adipose tissue, samples were extracted in dichloromethane/methanol. KH_2_PO_4_ was then added, and the solution centrifuged. The organic phase was evaporated under N_2_, with the resulting pellet dissolved in an isopropyl alcohol/hexane mixture. Individual lipid classes were separated by TLC using hexane-diethyl ether-acetic acid (70 : 30 : 1, *v*/*v*) as a solvent system, extracted from silica gel, and converted to FA methyl esters (FAME) using 1% sodium methoxide in dry methanol. FA profiles of phospholipid (PL) classes were established by gas chromatography using the Hewlett-Packard GC system, hydrogen as the carrying gas, a flame ionisation detector, and a carbowax-fused silica capillary column-DB-23 (60 m × 0.25 mm × 0.25 *μ*m; Agilent Technologies, USA). Individual FAME peaks were identified by comparing retention times with those of authentic standards (mix of FA standards, Restek, PA, USA) [15].

FADS activity was calculated based on FA composition in PLs as the product/precursor ratios reflecting activity of the following enzymes involved in FA metabolism: delta 5 desaturase (D5D) (20:5n3/20:4n3), delta 6 desaturase (D6D) (18:4n3/18:3n3), and delta 9 desaturase (D9D) (16:1n7/16 : 0, 18:1n9/18 : 0) [16].

### 2.4. Relative mRNA Expression

Total RNA was isolated from adipose tissue using RNA Blue (Top-Bio, Vestec, Czech Republic). The purity and concentration of RNA were determined using a NanoDrop spectrophotometer (NanoDrop™ 2000, ThermoFisher Scientific, Waltham, MA, USA). Reverse transcription and quantitative real-time PCR analysis were performed using the TaqMan RNA-to C_T_ 1-Step Kit, TaqMan Gene Expression Assay (ThermoFisher Scientific, Waltham, MA, USA), and the ViiA™ 7 Real-Time PCR System (ThermoFisher Scientific, Waltham, MA, USA). Relative expressions were determined after normalisation against *Hprt* as an internal reference and calculated using the 2^-*ΔΔ*Ct^ method. Results were run in triplicate.

### 2.5. Statistical Analysis

In this study, all data were analyzed using TIBCO Statistica™ 14.00 software (Prague, Czech Republic). We used one-way ANOVA to determine the effect of both MUFAs in HHTg rats compared to HHTg controls. To differentiate the specific effects of OA and POA, we applied LSD-Fisher's post hoc test for multiple comparison. All data was of normal distribution and expressed as the mean ± standard error of the mean (SEM). Statistical significance was set at *p* < 0.05.

## 3. Results

### 3.1. Effects of Oleic Acid and Palmitoleic Acid on Basal Metabolic Parameters and Circulating Inflammatory Markers

Supplementation with POA or OA in HHTg rats did not affect body weight or the relative weight of perirenal adipose tissue (PRAT) compared to the control group (Table 1). However, after OA administration, the relative weight of EAT significantly increased compared to HHTg controls. Neither of the MUFAs affected fasting glucose or AUC_0-180_. However, POA administration decreased nonfasting glycemia compared to the control group. Both MUFAs also significantly elevated glucagon and insulin levels compared to controls (Table 1).

In HHTg rats, the administration of both MUFAs affected serum lipid profiles (Table 1). Compared to controls, OA-treated HHTg rats exhibited significantly increased circulating levels of TG, but serum DAG, cholesterol, and HDL-cholesterol were not affected. On the other hand, POA administration markedly decreased DAG levels, while other circulating lipids remained unchanged compared to HHTg controls (Table 1).

As shown in Table 2, MUFA-treated HHTg rats exhibited reduced proinflammatory hsCRP, but TNF*α* and resistin remained unchanged. In addition, POA administration decreased proinflammatory IL-6 as well as MCP-1 serum levels. In contrast to POA, OA administration markedly increased concentrations of leptin compared to controls. OA supplementation slightly increased HMW adiponectin compared to HHTg controls. In the POA group, there was a major increase in HMW adiponectin along with elevated omentin levels, which may have contributed to the improvement of IS in peripheral tissues (Table 2).

### 3.2. Effects of Oleic Acid and Palmitoleic Acid on Lipolysis and NEFA Concentrations and Profiles

**Figure 1:**
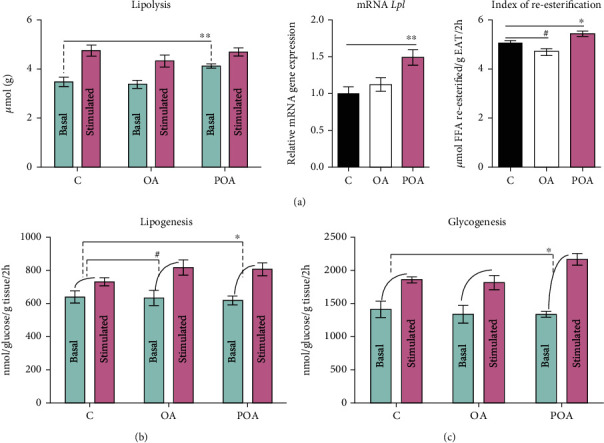
(a) The effect of oleic (OA) and palmitoleic acid (POA) on lipolysis, mRNA expression of lipoprotein lipase (*Lpl*), and reesterification of fatty acids (FA) in the adipose tissue expressed as NEFA/glycerol ratio; (b) the effect of OA and POA on insulin sensitivity (IS) in the adipose tissue expressed as basal and insulin-stimulated lipogenesis; (c) the effect of OA and POA on IS in the skeletal muscle expressed as basal and insulin-stimulated glycogenesis in hereditary hypertriglyceridemic (HHTg) rats. Data are expressed as means ± SEM; *n* = 8 for each experimental group. Differences were analyzed using one-way ANOVA and LSD-Fisher's *post hoc* test. ∗ denotes significance reflecting the effect of POA vs. C; # denotes significance reflecting the effect of OA vs. C; ^∗^*p* < 0.05, ^∗∗^*p* < 0.01, and ^#^*p* < 0.05.

**Figure 2:**
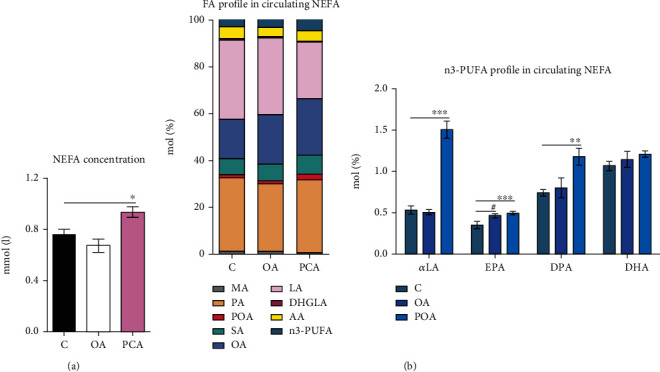
The effect of oleic (OA) and palmitoleic acid (POA) on NEFA concentrations (a) and fatty acid profile in circulating NEFA (b) in hereditary hypertriglyceridemic (HHTg) rats. Data are expressed as means ± SEM; *n* = 8 for each experimental group. Differences were analyzed using one-way ANOVA and LSD-Fisher's *post hoc* test. ∗ denotes significance reflecting the effect of POA vs. C; # denotes significance reflecting the effect of OA vs. C; ^∗^*p* < 0.05, ^∗∗^*p* < 0.01, ^∗∗∗^*p* < 0.001, and ^#^*p* < 0.05. MA: myristic acid; PA: palmitic acid; POA: palmitoleic acid; SA: stearic acid; OA: oleic acid; LA: linoleic acid; DHGLA: dihomo-*γ*-linoleic acid; AA: arachidonic acid; n3-PUFA: n3-polyunsaturated fatty acid; *α*LA: *α*-linoleic acid; EPA: eicosapentaenoic acid; DPA: docosapentaenoic acid; DHA: docosahexaenoic acid.

POA supplementation markedly increased basal lipolysis (Figure 1(a)) and mRNA gene expression of lipoprotein lipase (*Lpl*) in EAT (Figure 1(a)) compared to HHTg controls. These results, together with our findings of elevated FA reesterification in EAT (Figure 1(a)) and increased circulating NEFA levels (Figure 2(a)), indicate increased metabolic activity in adipose tissue compared to HHTg controls. In addition, POA administration positively influenced FA composition in circulating NEFA, as shown in Figure 2(b). The POA-treated group exhibited markedly elevated MUFA (*p* < 0.001; C—21.9 ± 0.3 mol%, OA—24.7 ± 0.7 mol%, and POA—27.7 ± 0.8 mol%) and n3-polyunsaturated fatty acids (n3-PUFA) profiles (*p* < 0.001; C—4.3 ± 0.1 mol%, OA—4.8 ± 0.2 mol%, and POA—5.8 ± 0.2 mol%) accompanied by a decreased n6-PUFA profile (*p* < 0.001; C—37.7 ± 0.5 mol%, OA—35.9 ± 0.9 mol%, and POA—30.1 ± 1.1 mol%), while SFA profile remained unchanged (C—36.2 ± 0.8 mol%, OA—34.6 ± 0.5 mol%, and POA—36.4 ± 0.9 mol%) compared to HHTg controls. For individual FA profiles in the NEFA lipid class, in the POA group, we observed significant increases in the profiles of POA (*p* < 0.01), OA (*p* < 0.001), *α*-linolenic acid (*α*LA), eicosapentaenoic acid (EPA), and docosapentaenoic acid (DPA) and decreases in the profiles of myristic acid (MA) (*p* < 0.001), linoleic acid (LA) (*p* < 0.001), and dihomo-*γ*-linolenic acid (DHGLA) (*p* < 0.001) compared to controls (Figure 2(b)).

In contrast to POA, OA supplementation resulted in slightly decreased FA reesterification in EAT, but circulating NEFA levels, mRNA gene expression of *Lpl*, and basal lipolysis in EAT were not affected compared to the HHTg control group (Figures 1(a) and 2(a)). OA administration generated a smaller increase in the MUFA profile (*p* < 0.01) of the NEFA lipid class. However, we observed major changes in individual FA profiles, specifically an increase in OA (*p* < 0.001) and EPA profiles accompanied by decreases in PA (*p* < 0.01), DHGLA (*p* < 0.001), and AA (*p* < 0.01) profiles compared to HHTg controls (Figure 2(b)).

### 3.3. Effects of Oleic and Palmitoleic Acid on Parameters of Insulin Sensitivity in Peripheral Tissues

**Figure 3:**
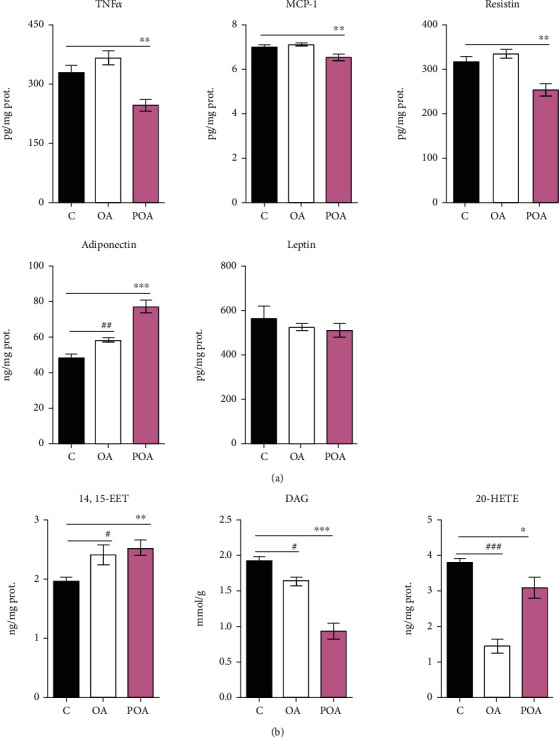
The effect of oleic (OA) and palmitoleic acid (POA) on secretion of cytokines and adipokines by adipose tissue (a) and the effect of OA and POA on the products of lipid metabolism, 14,15-epoxyeicosatrienoic acid (14,15-EET), diacylglycerols (DAG), and 20-hydroxyeicosatetraenoic acid (20-HETE) (b), in hereditary hypertriglyceridemic (HHTg) rats. Data are expressed as means ± SEM; *n* = 8 for each experimental group. Differences were analyzed using one-way ANOVA and LSD-Fisher's *post hoc* test. ∗ denotes significance reflecting the effect of POA vs. C; # denotes significance reflecting the effect of OA vs. C; ^∗^*p* < 0.05, ^∗∗^*p* < 0.01, ^∗∗∗^*p* < 0.001; ^#^*p* < 0.05, ^##^*p* < 0.01, and ^###^*p* < 0.001.

Supplementation with both MUFAs slightly increased insulin-stimulated lipogenesis (a reliable marker of improved IS in EAT) (Figure 1(b)) compared to HHTg controls. As shown in Figure 3(b), in contrast to OA, POA-treated rats exhibited markedly increased EAT levels of anti-inflammatory 14,15-EET compared to HHTg controls. In addition, after POA, we observed a major decrease in lipotoxic DAG and a slight decrease in proinflammatory AA-derived *α*-hydroxy metabolites (20-HETE) compared to the HHTg control group.

On the other hand, the OA-supplemented group showed only slightly reduced DAG and slightly increased 14,15-EET, while OA-treated rats exhibited markedly reduced proinflammatory 20-HETE compared to controls (Figure 3(b)). For EAT, there were no significant differences in protein content between the experimental groups (C—1.06 ± 0.05, OA—0.95 ± 0.02, and POA—1.04 ± 0.03).

**Figure 4:**
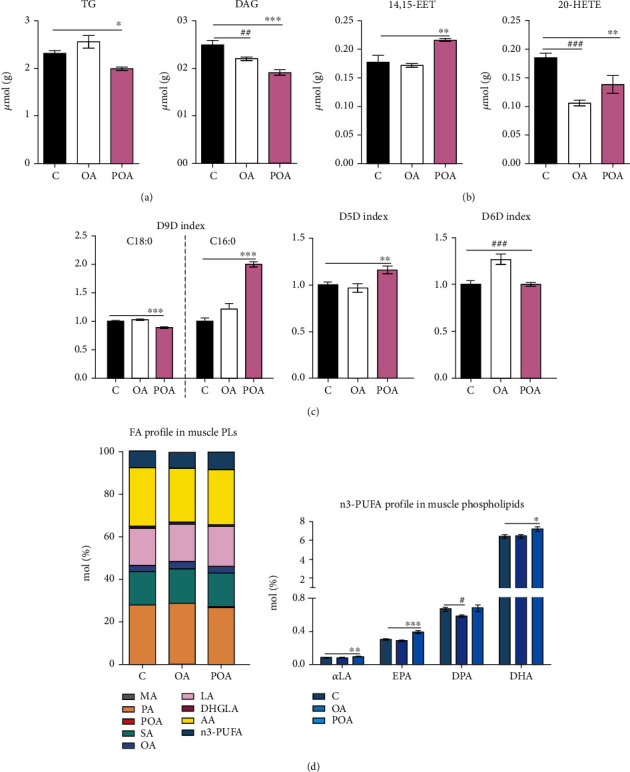
The effect of oleic (OA) and palmitoleic acid (POA) on concentration of lipids (a) and products of lipid metabolism (b) in the skeletal muscle in hereditary hypertriglyceridemic (HHTg) rats; (c) the effect of OA and POA on activity of desaturases (desaturase indexes) in the skeletal muscle of HHTg rats; (d) the effect of OA and POA supplementation on fatty acid profile of muscle membrane phospholipids (PLs) in HHTg rats. Data are expressed as means ± SEM; *n* = 8 for each experimental group. Differences were analyzed using one-way ANOVA and LSD-Fisher's *post hoc* test. ∗ denotes significance reflecting the effect of POA vs. C; # denotes significance reflecting the effect of OA vs. C; ^∗^*p* < 0.05, ^∗∗^*p* < 0.01, ^∗∗∗^*p* < 0.001, ^#^*p* < 0.05, ^##^*p* < 0.01, and ^###^*p* < 0.001. TG: triglycerides; DAG: diacylglycerols; 14,15-EET: 14,15-epoxyeicosatrienoic acid; 20-HETE: 20-hydroxyeicosatetraenoic acid; D9D: delta 9-desaturase; D5D: delta 5-desaturase; D6D: delta 6-desaturase; MA: myristic acid; PA: palmitic acid; POA: palmitoleic acid; SA: stearic acid; OA: oleic acid; LA: linoleic acid; DHGLA: dihomo-*γ*-linoleic acid; AA: arachidonic acid; n3-PUFA: n3-polyunsaturated fatty acid; *α*LA: *α*-linoleic acid; EPA: eicosapentaenoic acid; DPA: docosapentaenoic acid; DHA: docosahexaenoic acid; PL: phospholipid.

Only POA supplementation was associated with increased insulin-stimulated glycogenesis (a reliable marker of improved IS in the skeletal muscle) (Figure 1(c)), which was consistent with reduced accumulation of neutral TG as well as lipotoxic DAG (Figure 4(a)) in the skeletal muscle compared to the HHTg control group. After POA, the level of anti-inflammatory 14,15-EET in the skeletal muscle increased and proinflammatory 20-HETE decreased compared to controls (Figure 4(b)). In contrast, despite significant decreases after OA administration in the levels of muscle lipotoxic DAG and the proinflammatory AA metabolite 20-HETE (Figures 4(a) and 4(b)), IS in the skeletal muscle (Figure 1(c)), muscle TG, and 14,15-EET (Figures 4(a) and 4(b)) remained unchanged compared to HHTg controls.

### 3.4. Effects of Oleic Acid and Palmitoleic Acid on Inflammation and Lipid Profiles in Epididymal Adipose Tissue

The effects of POA administration were accompanied by a reduction in inflammatory cytokine levels in EAT. The POA-treated group exhibited significantly decreased levels of TNF*α*, MCP-1, and resistin in EAT (Figure 3(a)) compared to the control group. However, in the OA-supplemented group, EAT levels of MCP-1, TNF*α*, and resistin remained unchanged. In both MUFA-supplemented groups, EAT concentration of HMW adiponectin significantly increased, while leptin levels remained unchanged (Figure 3(a)).

**Figure 5:**
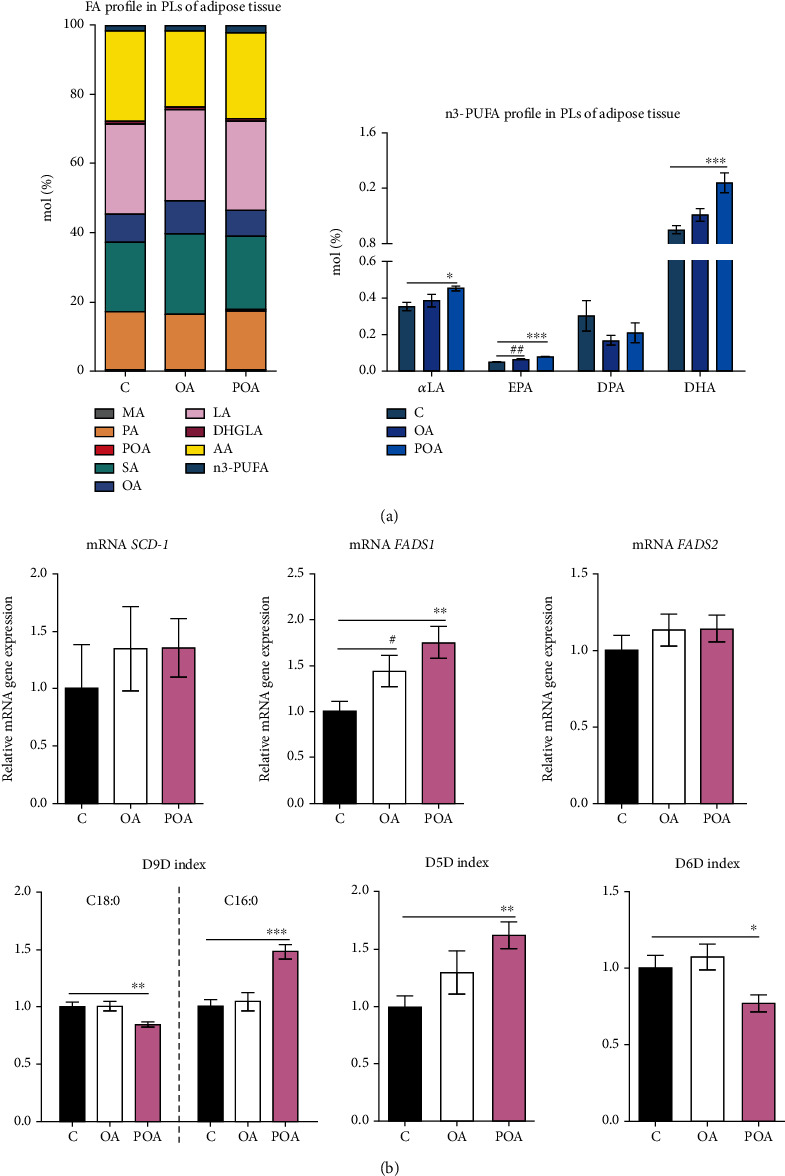
The effect of oleic (OA) and palmitoleic acid (POA) on fatty acid profile of adipose tissue membrane phospholipids (PLs) (a) and the effect of OA and POA on relative mRNA expression and activity (desaturation indexes) of desaturase system, SCD-1, FADS1, and FADS2 (b), in adipose tissue in hereditary hypertriglyceridemic (HHTg) rats. Data are expressed as means ± SEM; *n* = 8 for each experimental group. Differences were analyzed using one-way ANOVA and LSD-Fisher's *post hoc* test. ∗ denotes significance reflecting the effect of POA vs. C; # denotes significance reflecting the effect of OA vs. C; ^∗^*p* < 0.05, ^∗∗^*p* < 0.01, ^∗∗∗^*p* < 0.001; ^#^*p* < 0.05, and ^##^*p* < 0.01. MA: myristic acid; PA: palmitic acid; SA: stearic acid; LA: linoleic acid; DHGLA: dihomo-*γ*-linoleic acid; AA: arachidonic acid; n3-PUFA: n3-polyunsaturated fatty acid; *α*LA: *α*-linoleic acid; EPA: eicosapentaenoic acid; DPA: docosapentaenoic acid; DHA: docosahexaenoic acid; SCD: stearoyl-CoA desaturase; FADS: fatty acid desaturase; D9D: delta 9-desaturase; D5D: delta 5-desaturase; D6D: delta 6-desaturase; PL: phospholipid.

With regard to alterations in FA composition in EAT phospholipids, POA-supplemented rats had increased SFA (*p* < 0.05; C—35.2 ± 0.5 mol%, OA—37.5 ± 0.4 mol%, and POA—36.9 ± 0.5 mol%) and n3-PUFA (*p* < 0.01; C—1.8 ± 0.1 mol%, OA—1.9 ± 0.1 mol%, and POA—2.2 ± 0.1 mol%) profiles, while MUFA (C—10.3 ± 0.3 mol%, OA—11.7 ± 0.3 mol%, and POA—9.7 ± 0.2 mol%) and n6-PUFA profiles (C—52.8 ± 0.6 mol%, OA—48.9 ± 0.5 mol%, and POA—51.3 ± 0.5 mol%) remained unchanged compared to HHTg controls. OA-treated rats showed increased SFA (*p* < 0.01) and MUFA (*p* < 0.01) profiles and a decreased n6-PUFA (*p* < 0.001) profile compared to controls. As shown in Figure 5(a), for individual phospholipid FA profiles, after POA, we observed significant increases in the profiles of MA (*p* < 0.001), POA (*p* < 0.001), DHGLA (*p* < 0.001), *α*LA, EPA, and docosahexaenoic acid (DHA) and slight decrease in OA profile (*p* < 0.05) compared to controls. The OA-supplemented group exhibited a major decrease in the AA (*p* < 0.001) profile and increases in the profiles of MA (*p* < 0.05), POA (*p* < 0.05), stearic acid (SA) (*p* < 0.001), OA (*p* < 0.01), DHGLA (*p* < 0.001), and EPA compared to controls.

As shown in Figure 5(b), POA administration significantly altered mRNA gene expression and the activity indexes of desaturase enzymes in EAT phospholipids. After POA administration, the D9D activity index calculated from C18:0 fatty acids significantly decreased while the D9D activity index for C16:0 FA markedly increased compared to the HHTg control group. Relative mRNA gene expression for *SCD-1* remained unchanged. Although the D6D activity index significantly decreased in the POA-treated group, relative mRNA expression of *FADS2* was not affected in comparison with controls. The POA-treated HHTg group exhibited a markedly elevated activity index for D5D (associated with elevated relative mRNA expression of *FADS1*) compared to HHTg controls. In contrast, in the OA-treated group, except for slightly increased *FADS1* relative mRNA expression, we observed no changes in the activity indexes or relative mRNA expression of desaturase enzymes in EAT phospholipids compared to HHTg controls (Figure 5(b)).

### 3.5. Effects of Oleic Acid and Palmitoleic Acid on Lipids, Fatty Acid Profiles, and Fatty Acid Desaturases in the Skeletal Muscle

With regard to alterations in phospholipid FA profiles, POA-treated HHTg rats showed significantly elevated n3-PUFA (*p* < 0.01; C—8.2 ± 0.2 mol%, OA—8.1 ± 0.2 mol%, POA—9.1 ± 0.2 mol%) and MUFA (*p* < 0.05; C—6.7 ± 0.1 mol%, OA—7.1 ± 0.1 mol%, and POA—7.0 ± 0.1 mol%) profiles in the skeletal muscle compared to HHTg controls, while SFA (C—42.1 ± 0.2 mol%, OA—43.4 ± 0.4 mol%, and POA—41.3 ± 0.1 mol%) and n6-PUFA profiles (C—43.1 ± 0.2, OA—41.5 ± 0.3 mol%, and POA—42.6 ± 0.2 mol%) were not affected. On the other hand, the OA-treated group exhibited increased SFA (*p* < 0.01) and MUFA (p<0.01) profiles accompanied by a markedly reduced n6-PUFA profile (*p* < 0.001), with no change in the n3-PUFA profile.

For individual phospholipid FA compositions in the skeletal muscle, POA administration markedly increased the POA (*p* < 0.001) profile (Figure 4(d)). In addition, the POA-treated group exhibited markedly decreased PA (*p* < 0.05), MA (*p* < 0.01), OA (*p* < 0.001), and AA (*p* < 0.001) profiles compared to the control group. However, after POA administration, we observed significantly elevated SA (*p* < 0.05), LA (*p* < 0.001), DHGLA (*p* < 0.001), *α*LA, EPA, and DHA profiles. The OA-treated group showed increased MA (*p* < 0.001), PA (*p* < 0.05), POA (*p* < 0.05), SA (*p* < 0.05), and OA (*p* < 0.001) profiles accompanied by decreased DHGLA (*p* < 0.05), DPA, and AA (*p* < 0.001) profiles.

As shown in Figure 4(c), POA supplementation significantly altered the activity indexes for desaturase enzymes in muscle PLs compared to the HHTg control group. The POA-treated group exhibited a significantly elevated D5D activity index, which may be another indicator of improved muscle IS. Also, the POA-treated group exhibited a markedly reduced D9D activity index for C18:0 fatty acids accompanied by an elevated D9D activity index for C16:0 FA when compared to HHTg controls. The OA-treated group showed no changes in activity indexes for D5D and D9D in skeletal muscle PLs, but the activity index for D6D was markedly elevated (Figure 4(c)).

## 4. Discussion

In our study, we investigated the possible differential effects of POA and OA on lipid metabolism, inflammation, and IS in a nonobese prediabetic rodent model. OA administration produced a slight increase in circulating TG levels, but it was not associated with an increase in lipotoxic DAGs. Therefore, these results rather indicate increased mobilisation of lipid transport and deposition to EAT adipocytes serving as a protective barrier against the development of lipotoxicity [17], which was supported by a slight increase in EAT relative weight. On the other hand, in our study, POA supplementation had no effect on circulating serum TGs but markedly reduced the levels of lipotoxic DAGs, which can contribute to improved IS. Other studies have shown that POA can improve circulating lipid profiles in mice and humans. It should be noted, however, that in these studies, POA tended to have more of an effect on total lipids and cholesterol while TG levels remain largely unchanged [7]. OA is a major dietary FA and a major FA in TGs. Accordingly, the main transport medium is most likely a TG fraction that preferably incorporates OA, which supports our findings of slightly elevated circulating TG levels after OA administration.

Supplementation with POA led to a significant increase in circulating NEFA concentration and basal lipolysis; however, this was associated with a markedly beneficial qualitative alteration in FA profiles of the NEFA lipid class. This manifested in increased MUFA and, particularly, n3-PUFA profiles, accompanied by a decrease in n6-PUFA profiles. Only minor changes in NEFA composition were observed in the OA-treated group. Although elevated NEFA are sometimes associated with insulin-resistant states, many studies over several decades have demonstrated that elevated plasma NEFA levels are not necessarily linked to impaired IS [18]. Thus, increased NEFA together with beneficial changes in the qualitative profiles of these acids can play an important role in ameliorating IS after POA administration. Altered NEFA metabolism is understood to be pivotal in improving IS [19]. Individual circulating NEFA can act as molecule mediators that affect lipid and glucose metabolism, insulin signaling, and proinflammatory states, leading to inflammatory cytokine production in the adipose tissue [20]. Some studies have found an increase in lipolysis and a consequent rise in NEFA following POA supplementation [21], despite omitting an analysis of NEFA profiles. Increased lipolysis after POA supplementation is associated with greater metabolic activity in the adipose tissue, contributing to a reduction in visceral adiposity. It is also possible that the effect of POA on lipolysis is not dependent solely on insulin and therefore not necessarily directly related to circulating insulin levels. Indeed, it has been suggested that increased NEFA after POA is the result not only of elevated lipolysis but also of chylomicron-derived spill-over FA caused by the action of Lpl, which can also enrich the plasma NEFA pool [22].

Several studies have shown POA to be a modulator of TG metabolism, which can increase lipolysis associated with increased adipocyte Lpl activity, and found that OA has no effect on lipolysis or lipase gene expression despite significantly raising circulating TG levels [21]. The contrasting effects of OA and POA on Lpl may explain the differential accumulation of TG in the adipose tissue. POA supplementation has been shown to significantly increase *Lpl* mRNA gene expression, resulting in increased TG hydrolysis and reesterification of FAs in the adipose tissue followed by a greater release of NEFA into the circulation. These conditions may prevent TG accumulation in adipocytes, suggesting elevated metabolic activity in the adipose tissue. Relative to skeletal and cardiac muscle, in peripheral tissues such as the adipose tissue, Lpl functions as a rate-limiting enzyme in TG catabolism, thus regulating the influx and reesterification of FAs as well as, possibly, the amount of fat deposited [23]. In contrast, elevated dietary OA supplementation has been shown to have no effect on *Lpl* gene expression, with adipocytes unable to utilise the excess supply of FA. This results in increased storage of neutral TG in the adipose tissue, which is associated with slightly elevated adiposity. Lpl is a key enzyme in postprandial lipid metabolism, whose main site of action is the adipose tissue. Individual FAs, depending on quantity, chain length, or degree of saturation, may also influence Lpl activity [24]. In ob/ob mice with downregulated *Lpl* in VAT macrophages, TG deposition in adipose tissue macrophages decreased without effecting any reduction in total body weight or circulating TG levels. However, this was at the expense of elevated circulating NEFA levels and increased lipid deposition in nonadipose tissues [25].

In our study, POA supplementation increased insulin-stimulated lipogenesis, which is a reliable marker of adipose tissue IS. Improved IS was also observed after OA supplementation. Nevertheless, it is probable that these MUFA affect IS in different ways. Our results indicate that reducing inflammation can significantly improve IS in the adipose tissue. Low-grade inflammation is considered a key factor in the pathogenesis of IR, while decreased inflammation can delay the onset of T2DM [26, 27]. Substituting dietary MUFA for SFA activates beneficial anti-inflammatory mechanisms, reduces the development of dysregulations associated with diabetes and MS, and reverses the deleterious effect of SFA on peripheral tissues [28]. Fat composition in the diet is partially reflected in the FA profiles of cell membranes that induce IS modulation [29]. Recent animal and cell culture studies indicate that POA acts as a lipokine, which has the ability to alleviate chronic inflammation by modulating proinflammatory cytokine production and improving insulin signaling and lipid profiles [7]. OA acts differently by increasing mitochondrial *β*-oxidation, preventing inflammation, and activating various immunocompetent cells [30, 31]. These findings prove that the mechanisms and final effects of individual FAs can vary considerably.

According to our results, POA supplementation increased the n3-PUFA profile in the membrane PLs of the adipose tissue. An elevation in *α*LA, EPA, and DHA profiles in the PLs of the adipose tissue, which can subsequently affect cell signaling, has a positive effect on IS and membrane fluidity [32]. In our prediabetic HHTg rat model, POA increased IS in the adipose tissue, which was supported by an elevation in favorable adipokines (HMW adiponectin and omentin) and increased mRNA gene expression of *FADS1*, a gene correlated with IS by human studies [33]. In our study, mRNA gene expression of the desaturases *FADS2* and *SCD-1* was not affected following POA supplementation. In addition, increased D5D and D6D indexes pointed to a shift in metabolism favouring n3-PUFA. After POA supplementation, the D9D index of desaturated 18 : 0 significantly decreased, which may have been a compensatory mechanism for the increased D9D index of 16 : 0, which was markedly affected by the administration of POA, the product of desaturase reaction. One study focusing on elevation of circulating n3-PUFA found that EPA, DHA, and its metabolites improved IS, which was mediated by elevated adiponectin expression in the adipose tissue [34]. In agreement, our study confirmed that POA-induced elevation in circulating n3-PUFA profiles similarly improved IS. We also found an increase in the AA-derived anti-inflammatory metabolite 14,15-EET. Several studies have shown that EET act as mediators of insulin and glucagon secretion, affecting IS [35] as well as lipid metabolism [36]. Evidence that POA affects lipid metabolism was supported by markedly reduced levels of lipotoxic intermediates (DAG) in the adipose tissue in our POA-supplemented group of rats. Consistent with a study investigating the role of POA as a possible anti-inflammatory molecule [7], we found that POA-treated HHTg rats exhibited reduced proinflammatory cytokine concentrations in the adipose tissue, especially MCP-1, TNF*α*, and resistin.

On the other hand, the effect of OA in the adipose tissue mainly manifested in the displacement of membrane AA. In addition to a markedly reduced AA profile in membrane PLs, OA-supplemented HHTg rats exhibited markedly reduced proinflammatory AA-derived metabolites (20-HETE). Human and animal model studies have found that high levels of circulating 20-HETE are associated with obesity and MS [37] with adverse effects on insulin signaling and IS [38]. In our study, we found that OA administration in HHTg rats was associated with modulation of lipid metabolism and FA profiles in the membrane PLs of the adipose tissue. This resulted in increased insulin production and improved IS, supported by markedly increased HMW adiponectin concentrations and elevated mRNA gene expression of *FADS1* in the adipose tissue. Interestingly, none of the desaturase indexes were affected despite the administration of OA, a product of the desaturase reaction. This may have been due to the higher affinity of OA for the TG lipid layer.

One of the possible mechanisms by which OA contributes to inflammation is the activation of peroxisome proliferator-activated receptor gamma (PPAR*γ*), which is understood to mediate the reduction of TNF*α* production and stimulate insulin production [39]. Although our results confirmed greater insulin production after OA supplementation, TNF*α* concentrations in serum as well as in the adipose tissue were not affected. Notably, OA-induced modulation of lipid metabolism and FA profiles in the PL of the adipose tissue were more pronounced than the effect of POA on cytokine production. In HHTg rats, in addition to its marked effect on AA metabolism, OA may also have helped to decrease levels of lipotoxic DAG and slightly increase the anti-inflammatory mediator 14,15-EET. On the other hand, OA increased circulating leptin, which correlated with an increase in the relative weight of EAT. Increased insulin secretion may affect adipose tissue via a hormonal feedback loop, thus stimulating leptin secretion [40] and maintaining nutrient balance [41]. Several studies have confirmed that the distribution and reprogramming of lipid metabolism increases leptin production and glucagon-like peptide (GLP-1), resulting in improved IS in EAT [30].

In our study, the administration of POA and OA reduced levels of lipotoxic intermediate DAG in muscles. However, POA had a greater effect on lipotoxicity and the reduction of muscle TG levels compared to OA. These effects are understood to improve muscle IS after POA administration. Lipotoxic intermediates such as DAGs and ceramides aggravate endoplasmic reticulum stress, mitochondrial dysfunction, and ROS generation and impair insulin signaling [42]. Although AA profiles in muscle membrane PLs significantly decreased after administration of both MUFAs, the effects on AA metabolites were markedly different. While POA markedly increased anti-inflammatory EET, OA was more effective at reducing proinflammatory HETE levels in muscles. Our results demonstrate that in the skeletal muscle, POA increased incorporation of n3-PUFA into membrane PLs, possibly ameliorating IS in the skeletal muscle, whereas OA influenced AA metabolism and reduced lipotoxic intermediates. In the POA group, we observed a marked increase in the D5D index along with an elevation in the EPA profile in muscle PLs. These effects, which may improve insulin signaling and IS in muscle tissue, are understood to apply to nonobese individuals and be independent of the presence of obesity [43].

**Figure 6:**
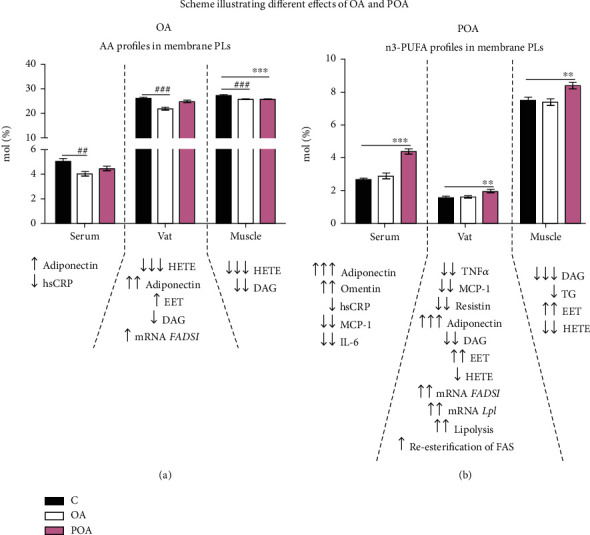
Final scheme of different putative involvement of oleic (OA) and palmitoleic (POA) acid on metabolic effects in peripheral tissues associated with insulin sensitivity (IS) improvement and inflammatory amelioration. OA supplementation (a) in hereditary hypertriglyceridemic (HHTg) rats affected mainly n6-polyunsaturated fatty acid (n6-PUFA) metabolism—especially arachidonic acid (AA) metabolism. OA-treated rats exhibited not only decreased AA profiles but also decreased proinflammatory AA-derived metabolites (HETEs) in peripheral tissues, which could ultimately reduce chronic inflammation. Slightly increased fatty acid desaturase (*FADS1*) gene expression after OA along with increased adiponectin production by adipose tissue was reflected in slightly improved IS of visceral adipose tissue (VAT). On the other hand, POA supplementation (b) in HHTg rats mainly affected the incorporation of n3-PUFA into membrane phospholipids of the skeletal muscle and VAT and markedly decreased toxic lipid accumulation into peripheral tissues, which could contribute to increased membrane fluidity. Considerably increased *FADS1* gene expression together with elevated adiponectin production by VAT led to a significant increase in circulating adiponectin and omentin levels. These alterations could contribute to improvement of IS of both muscle and VAT. In addition to the insulin-sensitising effect, POA-treated rats showed markedly decreased proinflammatory cytokine production by VAT. All these changes in the adipose tissue, together with markedly elevated basal lipolysis, gene expression of *Lpl*, and fatty acid (FA) reesterification, indicate a significant increase in metabolic activity of VAT and lead to the designation of POA as a lipokine. Data are expressed as means ± SEM; *n* = 8 for each experimental group. Differences were analyzed using one-way ANOVA and LSD-Fisher's *post hoc* test. ∗ denotes significance reflecting the effect of POA vs. control (c); # denotes significance reflecting the effect of OA vs. C; ↑ denotes significance reflecting the effect of OA/POA therapy vs. the C group. ^∗∗^*p* < 0.01, ^∗∗∗^*p* < 0.001, ^##^*p* < 0.01, ^###^*p* < 0.001, ^↑^*p* < 0.05, ^↑↑^*p* < 0.01, and ^↑↑↑^*p* < 0.001.

Based on the above findings, we speculate that OA has a beneficial effect on lipid metabolism and slightly improves chronic inflammation via modulation of AA metabolism, whereas POA activates basal lipolysis and reesterification, elevates Lpl activity, modulates n3-PUFA metabolism in membrane PLs, and markedly affects pro- and anti-inflammatory cytokine production by EAT. Thus, POA acts as a lipokine capable of influencing and modulating metabolic processes in adipose and other peripheral tissues by altering cytokine secretion and modulating circulating NEFA profiles (Figure 6).

## 5. Conclusions

Our results demonstrate that supplementation with both MUFAs ameliorated IS in peripheral tissues and modulated inflammation in a prediabetic model. However, the mechanisms behind these improvements and the extent of their effects were different. While POA served as a lipokine affecting IS and cytokine secretion, OA modulated AA metabolism. The mechanisms behind the positive effects of POA may involve FA profile alterations to adipose tissue and skeletal muscle membrane PLs as well as circulating NEFA, leading to a marked increase in n3-PUFA profiles. The positive changes to IS in the adipose tissue we observed may also be attributable to adipocytokine production and an increase in the anti-inflammatory metabolites of n3-PUFA. Also, the mechanism that OA deploys to ameliorate IS is associated with decreased proinflammatory AA-derived proinflammatory *α*-hydroxy metabolites (20-HETE).

## Figures and Tables

**Table 1:** Basal metabolic and morphological characteristics of hereditary hypertriglyceridemic (HHTg) rats after oleic acid (OA) and palmitoleic acid (POA) administration.

	C	OA	POA	*p*_ANOVA_	*p*_OA/POA_
Body weight (g)	415 ± 9	433 ± 8	421 ± 5	n.s.	
EAT weight (g/100 g BW)	1.70 ± 0.06	2.00 ± 0.06^##^	1.87 ± 0.08	<0.05	n.s.
PRAT weight (g/100 g BW)	2.32 ± 0.07	2.57 ± 0.06	2.35 ± 0.12	n.s.	
Serum TG (mmol/l)	3.01 ± 0.15	3.63 ± 0.09^##^	3.36 ± 0.14	<0.01	<0.05
Serum DAG (mmol/l)	0.063 ± 0.005	0.072 ± 0.004	0.046 ± 0.004^∗^	<0.01	<0.001
Serum CHOL (mmol/l)	1.94 ± 0.05	1.94 ± 0.04	1.93 ± 0.05	n.s.	
HDL-C (mmol/l)	0.98 ± 0.04	0.93 ± 0.02	0.89 ± 0.01	n.s.	
Fasting glucose (mmol/l)	6.3 ± 0.2	6.2 ± 0.1	6.4 ± 0.1	n.s.	
Non-fasting glucose (mmol/l)	8.3 ± 0.1	8.5 ± 0.1	8.0 ± 0.1^∗^	<0.01	<0.01
AUC_0-180_	1519 ± 27	1457 ± 25	1504 ± 42	n.s.	
Insulin (nmol/l)	0.149 ± 0.010	0.190 ± 0.015^#^	0.221 ± 0.017^∗∗^	<0.01	n.s.
Glucagon (pg/ml)	161.1 ± 4.6	180.1 ± 6.3^#^	187.1 ± 6.9^∗∗^	<0.05	n.s.

Data are expressed as means ± SEM; *n* = 8 for each experimental group. Differences were analyzed using one-way ANOVA and LSD-Fisher's *post hoc* test. *P*_ANOVA_ denotes the significance of oleic acid (OA)/palmitoleic acid (POA) supplementation vs. the control (C) group; *P*_OA/POA_ denotes the significance reflecting the effect of OA vs. POA. ∗ denotes significance reflecting the effect of POA vs. C; # denotes significance reflecting the effect of OA vs. C; ^∗^*p* < 0.05, ^∗∗^*p* < 0.01, ^#^*p* < 0.05, and ^##^*p* < 0.01.

**Table 2:** Circulating inflammatory markers and adipocytokines in hereditary hypertriglyceridemic (HHTg) rats after oleic acid (OA) and palmitoleic acid (POA) administration.

	C	OA	POA	*p*_ANOVA_	*p*_OA/POA_
HMW adiponectin (*μ*g/ml)	5.19 ± 0.20	5.80 ± 0.11^#^	6.59 ± 0.24^∗∗∗^	<0.001	<0.01
hsCRP (mg/ml)	1.34 ± 0.03	1.13 ± 0.07^#^	1.14 ± 0.07^∗^	<0.05	n.s.
MCP-1 (ng/ml)	7.02 ± 0.10	7.13 ± 0.07	6.55 ± 0.15^∗∗^	<0.01	<0.01
TNF*α* pg/ml	2.45 ± 0.12	2.55 ± 0.19	2.65 ± 0.14	n.s.	
Leptin (pg/ml)	8.33 ± 0.43	11.34 ± 0.48^###^	8.29 ± 0.45	<0.001	<0.001
Omentin (ng/ml)	2.59 ± 0.22	2.68 ± 0.14	3.42 ± 0.24^∗∗^	<0.05	<0.05
Resistin (ng/ml)	51.14 ± 1.02	53.02 ± 0.83	53.35 ± 1.12	n.s.	
IL-6 (pg/ml)	133.23 ± 6.19	134.44 ± 7.73	102.31 ± 7.81^∗∗^	<0.01	<0.01

Data are expressed as means ± SEM; *n* = 8 for each experimental group. Differences were analyzed using one-way ANOVA and LSD-Fisher's *post hoc* test. *p*_ANOVA_ denotes the significance of oleic acid (OA)/palmitoleic acid (POA) supplementation vs. the control (C) group; *p*_OA/POA_ denotes the significance reflecting the effect of OA vs. POA. ∗ denotes significance reflecting the effect of POA vs. C; # denotes significance reflecting the effect of OA vs. C; ^∗^*p* < 0.05, ^∗∗^*p* < 0.01, ^∗∗∗^*p* < 0.001, ^#^*p* < 0.05, and ^###^*p* < 0.001.

## Data Availability

The data used to support the findings of this study are included within the article.

## Abbreviations

AA: Arachidonic acid
DAG: Diacylglycerol
D5D: Delta 5-desaturase
D6D: Delta 6-desaturase
D9D: Delta 9-desaturase
EAT: Epididymal adipose tissue
EET: Epoxyeicosatrienoic acid
FA: Fatty acid
FADS: Fatty acid desaturase
HETE: Hydroxyeicosatetraenoic acid
hsCRP: High-sensitivity C-reactive protein
IL-6: Interleukin 6
IS: Insulin sensitivity
Lpl: Lipoprotein lipase
MCP-1: Monocyte chemoattractant protein-1
MS: Metabolic syndrome
MUFA: Monounsaturated fatty acid
NEFA: Nonesterified fatty acid
OA: Oleic acid
PL: Phospholipid
POA: Palmitoleic acid
PPAR*γ*: Peroxisome proliferator-activated receptor *γ*
PUFA: Polyunsaturated fatty acid
ROS: Reactive oxygen species
SCD: Stearoyl-coenzyme A desaturase
SFA: Saturated fatty acid
TG: Triglyceride
TNF*α*: Tumour necrosis factor *α*
VAT: Visceral adipose tissue.
